# Acknowledgement to Reviewers of *Journal of Personalized Medicine* in 2017

**DOI:** 10.3390/jpm8010005

**Published:** 2018-01-15

**Authors:** Journal of Personalized Medicine Editorial Office

**Affiliations:** MDPI AG, St. Alban-Anlage 66, 4052 Basel, Switzerland

Peer review is an essential part in the publication process, ensuring that *Journal of Personalized Medicine* maintains high quality standards for its published papers. In 2017, a total of 22 papers were published in the journal. Thanks to the cooperation of our reviewers, the median time to first decision was 31.5 days and the median time to publication was 64.5 days. The editors would like to express their sincere gratitude to the following reviewers for their time and dedication in 2017:

Albani, DiegoKonstantinidou, ValentiniAlyass, AkramKukreja, MuskanAntoñanzas, FernandoKumar, AmbujBarnett, MatthewKuo, Grace M.Benedetti, SaraKwiecien, RobertBenson, MikaelLawson, WilliamBonetti, MarcoLe Faucheur, AlexisBorchers, ChristophLebo, Matthew S.Brownstein, Catherine A.Lee, Su-JunCantor, MichaelLewis, Joshua P.Chadwick, RuthLima, John J.Codacci Pisanelli, GiovanniMacKay, DylanDel-Aguila, JorgeMaeda, HiroshiDemirev, PlamenMaiolo, DanieleDorado, PedroManolopoulos, Vangelis G.Drasler, BarbaraMarshall, DeborahDrayna, DennisMartino, SabataDressler, Lynn G.Merkel, Olivia M.Enderling, HeikoMoniri, Nader H.Ethgen, OlivierMoulton, HongFeeney, EmmaMustacchi, GiorgioFernández-Santander, AnaOlson, JanetFilippelli, AmeliaOrlando, Lori A.Fontaine-Bisson, BénédicteOverby, CaseyGaedigk, AndreaPalamà, IlariaGarcia-Bailo, BibianaPark Sung-Shim, LaniGarrison, LouisPatrinos, George P.Gibson, GregPhillips, Kathryn A.Grebien, FlorianPoulymenopoulou, MikaelaGuerra-Rebollo, MartaPrainsack, BarbaraHart, StevenRahm, AlannaHertz, Daniel L.Ramsey, LauraHiratsuka, MasahiroRogowski, WolfHoppu, UllaShvartsman, StanislavHourigan, ChristopherThomas, CourtneyIJzerman, Maarten J.Vears, DanyaJanowski, MiroslawVera-González, JulioJenab, MazdaVesin, Jean-MarcJoore, ManuelaWatanabe, MasatoshiKanai, MasashiWu, Alan HbKarazniewicz-Lada, MartaYamakawa-Kobayashi, KimikoKarnes, Jason H.Yin, JunKimokoti, RuthZelikin, Alexander N.Kisor, David F.Ziegelstein, Roy C.

